# Association between cerebral small vessel disease and malnutrition risk: a retrospective cross-sectional study

**DOI:** 10.3389/fneur.2025.1512109

**Published:** 2025-06-18

**Authors:** Junyi Liu, Jie Lin, Jieying Zhuang, Qian Luo, Huijuan Wang, Ruiyan Xiao, Xudong Yang, Jiangping Cai

**Affiliations:** ^1^The School of Clinical Medicine, Fujian Medical University, Fuzhou, China; ^2^Department of Neurology, The First Hospital of Quanzhou Affiliated to Fujian Medical University, Quanzhou, China; ^3^The Graduate School of Fujian Medical University, Fuzhou, China

**Keywords:** cerebral small vessel disease, malnutrition risk, imaging markers, geriatric nutritional risk index, controlling nutritional status

## Abstract

**Introduction:**

Malnutrition is associated with increased morbidity and mortality from multiple diseases. However, the relationship between cerebral small vessel disease (CSVD) and malnutrition or malnutrition risk remains underexplored. This retrospective study investigated the association between malnutrition risk and CSVD, along with its common imaging markers.

**Methods:**

A total of 806 participants from a neurology department underwent cranial MRI scans and nutritional assessments. The presence of imaging markers of CSVD, including white matter hyperintensities, lacune, perivascular spaces, and cerebral microbleeds, was evaluated by expert neurologists. Malnutrition risk was assessed using the Geriatric Nutritional Risk Index (GNRI) and Controlling Nutritional Status (CONUT) scores. Logistic regression, subgroup, and interaction analyses were performed to evaluate the associations between malnutrition risk, CSVD, and its common imaging markers.

**Results and discussion:**

After adjusting for potential confounders, patients at risk of malnutrition, as identified by both the GNRI and CONUT scores, exhibited more severe CSVD and its common imaging markers. Further analyses revealed interactions between GNRI score and smoking, highlighting potential modifying effects on the relationship between malnutrition risk and CSVD. Collectively, malnutrition risk, as assessed by objective nutritional indices, is independently associated with CSVD and its common imaging markers. These results emphasize the importance of addressing malnutrition in the prevention and management of CSVD.

## 1 Introduction

Cerebral small vessel disease (CSVD), as the primary cause of vascular dementia, can result in a significant decline in cognitive function, gait, and balance (1). It contributes to 25% of ischemic strokes (2). The early stages of CSVD are frequently asymptomatic and challenging to diagnose clinically, underscoring the importance of neuroimaging in the early detection and management of the disease. Cranial MRI is a key modality for assessing CSVD. The typical neuroimaging manifestations of CSVD include white matter hyperintensities (WMH) of presumed vascular origin, perivascular spaces (PVS), lacune (also of presumed vascular origin), recent subcortical small infarcts cerebral microbleeds (CMB), and brain atrophy (3).

Malnutrition is a multifaceted disease characterized by a reduction in body fat and/or muscle mass resulting from inadequate nutrient intake or absorption (4). It has detrimental effects on daily activities, leading to a decline in the quality of life and physical functioning (5). Moreover, malnutrition is associated with increased morbidity and mortality from multiple diseases (6). On a broader scale, the recurrent hospitalizations and prolonged stays caused by malnutrition pose a significant financial burden on the healthcare system (7).

The diagnostic criteria for malnutrition established by the European Society for Clinical Nutrition and Metabolism (ESPEN) (8) and the Global Leadership Initiative on Malnutrition (GLIM) (9) are widely applied in clinical practice. However, these criteria do not include an assessment of malnutrition risk. Several objective nutritional indicator assessment tools have been developed and effectively applied, such as the Geriatric Nutritional Risk Index (GNRI) (10) and the Controlling Nutritional Status (CONUT) (11). The GNRI and CONUT have demonstrated good accuracy and predictive capability in evaluating the risk of moderate to severe malnutrition (12, 13).

Malnutrition can affect individuals of all age groups. However, elderly people (≥65 years) are particularly susceptible due to a combination of risk factors (14). Currently, it is estimated that approximately one-quarter of elderly individuals are either malnourished or at risk of malnutrition (15). Furthermore, the incidence of CSVD tends to increase with age (16). However, studies examining the relationship between CSVD and malnutrition risk are currently limited.

In this study, we aimed to investigate the association between CSVD and malnutrition risk, assessed using GNRI and CONUT. Our findings suggest the importance of improving the nutritional status of the population with CSVD.

## 2 Methods

### 2.1 Study participants

This retrospective cross-sectional study recruited patients who were either outpatients or inpatients at the Department of Neurology, Quanzhou First Affiliated Hospital of Fujian Medical University from September 2020 to December 2024. Inclusion criteria: (1) Age ≥18 years old; (2) Patients underwent 3.0T cranial MRI scans, including T1-weighted imaging (T1WI), T2-weighted imaging (T2WI), Fluid Attenuated Inversion Recovery (FLAIR), Diffusion Weighted Imaging (DWI) and Susceptibility Weighted Imaging (SWI), within 7 days of hospitalization; (3) Patients received blood routine, routine biochemistry, and other related tests within 24 h of treatment or hospitalization. Exclusion criteria: (1) Patients with acute cerebral infarction with high signal intensity lesions on DWI and diameter >20 mm, history of large-area cerebral infarction due to large vessel occlusion, or conditions that hindered the diagnosis of CSVD; (2) Patients with severe stenosis and occlusion of large vessels in the head or neck on computed tomography angiography or digital subtraction angiography; (3) Patients with acute cerebral hemorrhage, acute subarachnoid hemorrhage, or history of cerebrovascular malformation or aneurysmal subarachnoid hemorrhage; (4) Patients with definite non-vascular white matter lesions, such as multiple sclerosis, adult white matter dysplasia, metabolic encephalopathy, etc.; (5) Patients who suffered from severe organic diseases, such as severe liver and kidney dysfunction, malignant tumors (especially chronic lymphocytic leukemia), etc.; (6) Patients with recent infections within the past 2 weeks; (7) Patients with incomplete clinical data. This study was approved by the Ethics Committee of the First Hospital of Quanzhou (approval No. [2020] 168). Informed consent was obtained from all participants, ensuring that their private data remained anonymous and confidential.

### 2.2 Clinical data collection

Demographic and clinical characteristics of patients were collected upon admission, including blood routine results, blood biochemistry results, hypertension, diabetes mellitus, stroke history, coronary heart disease, dyslipidemia, current smoking history, current alcohol consumption, medication use (antiplatelets and statins), etc.

### 2.3 MRI and image analysis

Patients received plain MRI scans, including T1WI, T2WI, FLAIR, DWI, and SWI sequences, using a 3.0T MRI scanner (Signa, GE Healthcare, Milwaukee, WI, USA), equipped with an 8-channel head-neck combined coil. To minimize motion artifacts, a foam pillow secured the head of each participant within the coil. The imaging parameters for each sequence were as follows: T1WI, repetition time (TR) of 6.67 ms, echo time (TE) of 2.99 ms, flip angle (FA) of 8.0°, field of view (FOV) of 240 × 240 mm^2^, slice thickness of 1.0 mm, slice gap of 0.5 mm, matrix of 240 × 240, and number of excitations (NEX) of 1; T2WI, TR of 4000 ms, TE of 115.76 ms, FA of 90.0°, FOV of 230 × 230 mm^2^, slice thickness of 5.0 mm, slice gap of 6.0 mm, and NEX of 1; FLAIR, TR of 4800 ms, TE of 340 ms, FA of 90.0°, FOV of 250 × 250 mm^2^, slice thickness of 2.0 mm, slice gap of 1.0 mm, matrix of 224 × 223, and NEX of 1; DWI, TR of 2688.11 ms, TE of 74.90 ms, FA of 90.0°, FOV of 230 × 230 mm^2^, slice thickness of 5.0 mm, slice gap of 6.0 mm, matrix of 128 × 114, and NEX of 1; and, SWI, TR of 31 ms, FA of 17.0°, FOV of 230 × 230 mm^2^, slice thickness of 15.0 mm, slice gap of 1.0 mm, and NEX of 1.

Two expert neurologists, each with 10 years of clinical experience, independently analyzed the cranial MRI scans of the patients, blinded to baseline data. In cases of disagreement, the two neurologists discussed until reaching a consensus or consulted a third experienced radiologist, who was also blinded to baseline data. The evaluation of WMH, lacune, PVS, and CMB was conducted following the standards for reporting vascular changes on neuroimaging-2 (3). WMH was defined if there was high signal intensity on T2WI or FLAIR and iso- or hypo-intensity on T1WI. The severity grading of WMH was primarily based on the Fazekas scale (17), with separate assessments for periventricular WMH (PWMH) and deep WMH (DWMH). The study population was categorized into mild (0–2 points) and moderate to severe (3–6 points) groups based on the severity of the total burden of WMH, calculated as the cumulative score of PWMH and DWMH on a scale of 0–6 points. Subsequently, the WMH severity at different locations was assessed, with PWMH and DWMH classified into mild (0–1 point) and moderate to severe (2–3 points) categories. PVS showed a resemblance to vascular tracts with well-defined borders, appearing linear on axial sections and oval or round on longitudinal sections, with high signal intensity on T2WI and low signal intensity on T1WI. PVS generally have a diameter of < 3 mm, and differentiation from lacunar infarction and WMH is necessary when their diameter is >3 mm. FLAIR sequences are helpful in this distinction. We also examined basal ganglia PVS (BG-PVS) and centrum semiovale PVS (CSO-PVS), calculated the number of PVS in one hemisphere at the most severely affected level in various brain regions, and assigned grades according to specific criteria (18): grade 0 for no PVS, grade 1 for ≤ 10 PVS, grade 2 for 11–20 PVS, grade 3 for 21–40 PVS, and grade 4 for >40 PVS. Based on the PVS grades, the study cohort was divided into a mild group (PVS ≤ 10) and a moderate to severe group (PVS > 10) (19). The quantification of PVS at various sites led to the classification of PVS burden in the BG and CSO into mild (PVS ≤ 10) and moderate to severe (PVS > 10) groups. Lacune was defined as round or oval hyperintensities on T2WI sequences, with diameters ranging from 3 to 15 mm, predominantly found in subcortical, thalamic, and basal ganglia regions. Their signal intensity was similar to cerebrospinal fluid, showing hypointense on T1WI, centrally hypointense on FLAIR sequences, and surrounded by hyperintense rings. CMB was defined as rounded, hypodense lesions with sizes of 2–10 mm in SWI sequences. The total CSVD score (0–4 points) was calculated based on the presence of WMH, lacune, PVS, and CMB, with 1 point each for WMH burden (PWMH 3 points and/or DWMH 2–3 points), presence of lacune, moderate-to-severe BG-PVS (*N* > 10), and presence of CMB (16).

### 2.4 Malnutrition risk evaluation

The GNRI score was calculated as [1.489 × serum albumin (g/L) + 41.7 × current body weight (kg)/ideal body weight (kg)] (10). If the current weight exceeded the ideal weight, the current weight (kg)/ideal weight (kg) was set to 1. The ideal weight was determined using the Lorentz formula (20), which was as follows: for males, ideal weight = height (cm) – 100 – [(height cm – 150)/4]; and for females, ideal weight = height (cm) – 100 – [(height cm – 150)/2.5]. GNRI scores greater than 98, 92 to 98, 82 to 91, and less than 82 were considered indicative of normal, mild, moderate, and severe malnutrition risk, respectively.

The CONUT evaluated the risk of malnutrition score based on serum albumin, total cholesterol, and total lymphocyte count (11). Scores ranging from 0 to 1, 2 to 4, 5 to 8, and 9 to 12 were categorized as representing normal, mild, moderate, and severe malnutrition risk levels. The moderate and severe malnutrition status was combined into moderate-severe malnutrition according to the previous description (21).

### 2.5 Statistical analyses

Statistical analyses were conducted using R version 4.4.2 (R Foundation for Statistical Computing, Vienna, Austria) and GraphPad Prism 8 (GraphPad Software Inc., San Diego, CA, USA). The categorical variables are presented as percentages and compared with χ^2^ test or Fisher's exact test. The continuous variables are expressed as medians with interquartile ranges or means with standard deviations and analyzed with the Mann–Whitney *U* test or Student's *t*-test. Univariate and multivariate logistic regression analyses were utilized to identify factors influencing CSVD and its imaging markers. In multivariate logistic regression model 1, the age and sex were adjusted. The multivariate logistic regression model 2 was based on model 1 and the factors of systolic blood pressure, body mass index (BMI), history of stroke, hypertension, diabetes, coronary heart disease, dyslipidemia, smoking, alcohol consumption, laboratory parameters (neutrophil-to-lymphocyte ratio, hemoglobin A1c, homocysteine, and estimated glomeru lar filtration rate), and medication use (antiplatelets and statins) were adjusted. Additionally, subgroup analyses and interaction tests were conducted to assess the association between GNRI, CONUT, and CSVD across different subgroups. All statistical tests were two-tailed, and significance was considered for *P* < 0.05.

## 3 Results

### 3.1 Baseline characteristics

**Figure 1:**
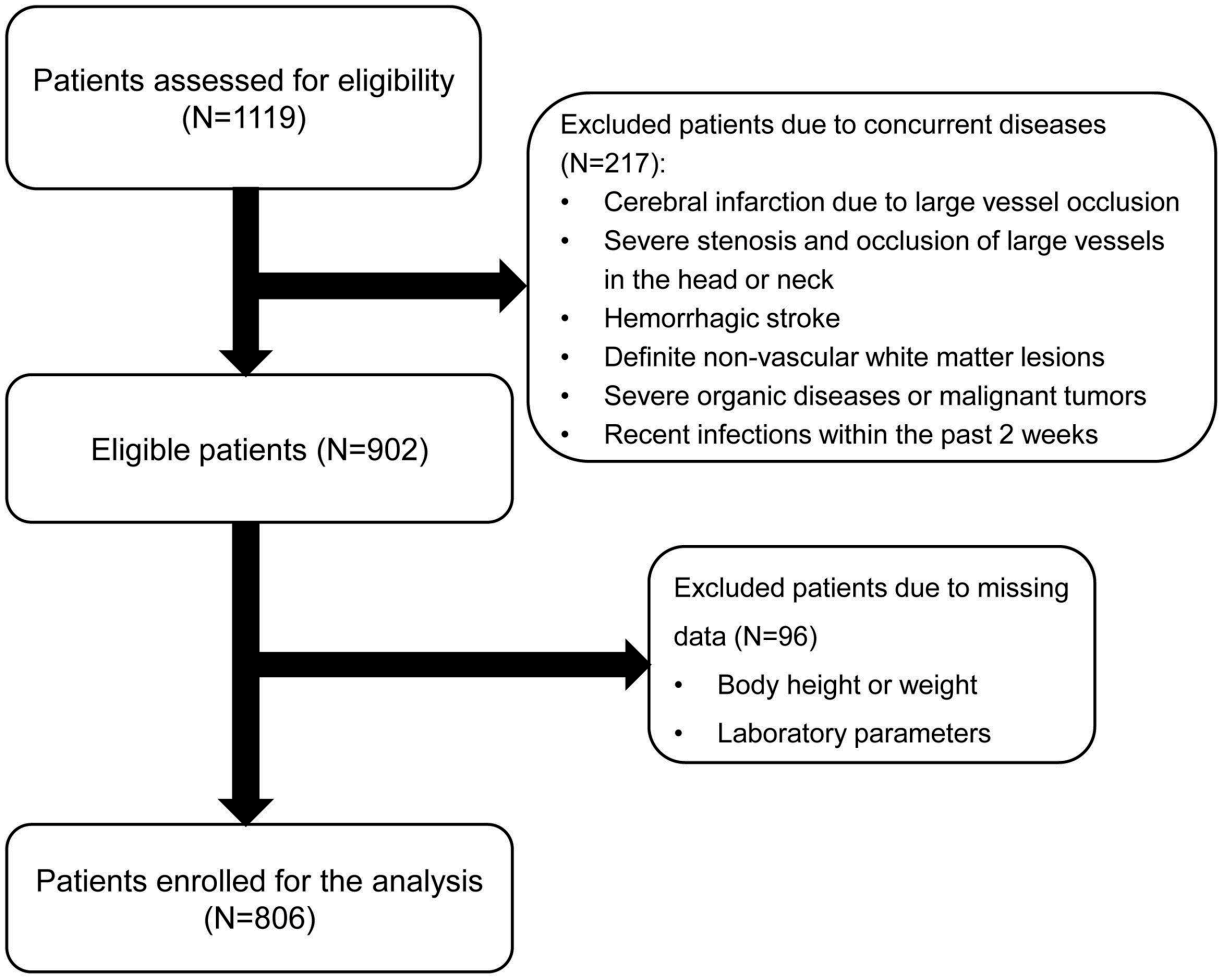
Flow chart of patient enrollment.

Initially, 1,119 participants were enrolled in the study. After excluding individuals with cerebral infarction, severe stenosis and occlusion of large vessels in the head or neck, cerebral hemorrhage, missing laboratory tests or clinical data, recent infection history within 2 weeks, and cancer, a total of 806 patients were included for analysis (Figure 1). Their baseline characteristics are presented in Supplementary Table 1. Based on the total CSVD score, the patients were grouped into the non-CSVD group (total CSVD score = 0) (*n* = 450) and the CSVD group (total CSVD score ≥ 1) (*n* = 356). Patients in the CSVD group were older, predominantly male, and exhibited a higher prevalence of hypertension, diabetes, coronary heart disease, history of stroke, dyslipidemia, and current smoking (*P* < 0.05). Furthermore, they demonstrated elevated levels of systolic blood pressure, diastolic blood pressure, neutrophil count, neutrophil-to-lymphocyte ratio, hemoglobin A1c, and homocysteine (*P* < 0.05). Additionally, a larger proportion of patients in the CSVD were prescribed medications compared to those in the non-CSVD group (*P* < 0.05). Notably, the CSVD group also presented higher rates of mild and moderate-to-severe malnutrition risk, as assessed by GNRI and CONUT scores, than the non-CSVD group (*P* < 0.05).

### 3.2 Malnutrition risk according to GNRI and CONUT scores

**Figure 2:**
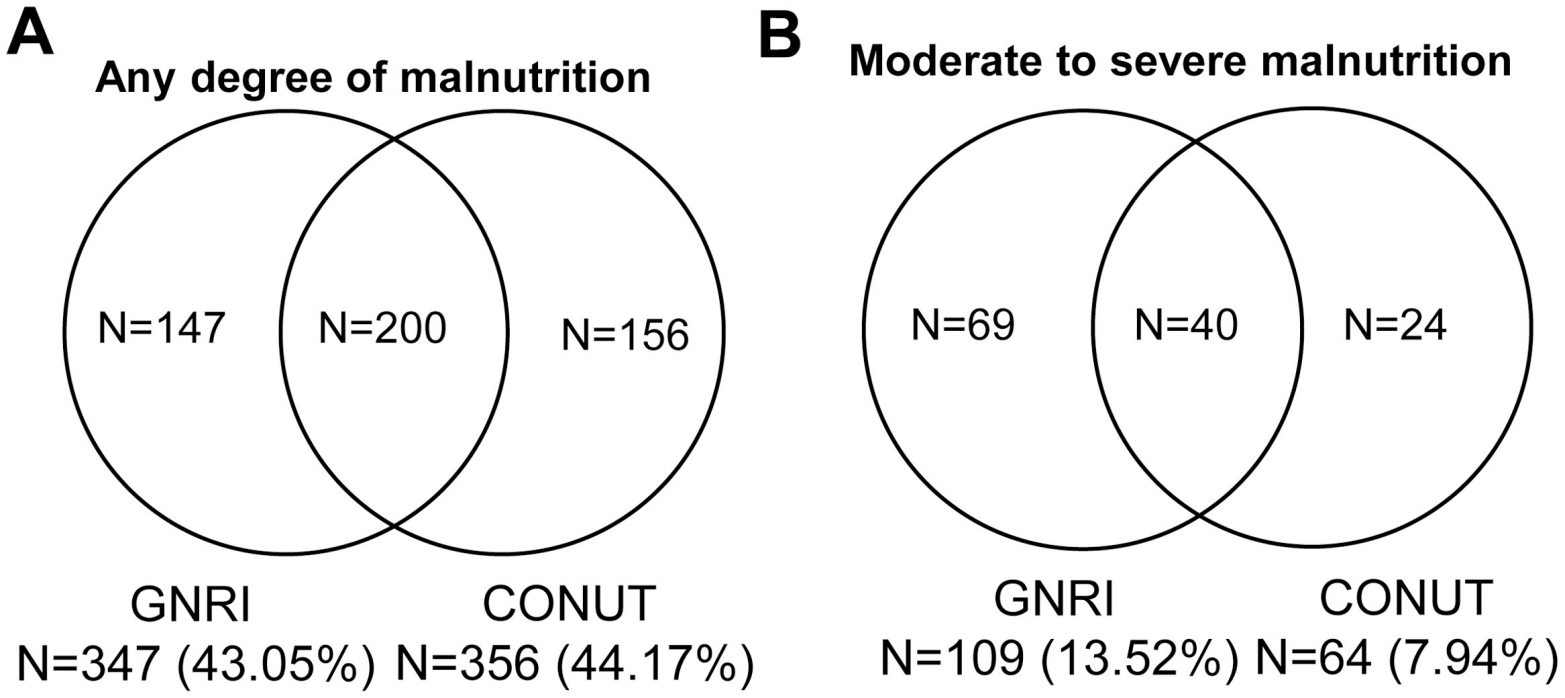
Venn diagram of malnutrition risk assessed by GNRI and CONUT. **(A)** Malnutrition risk as identified by the GNRI and CONUT. **(B)** Moderate to severe malnutrition risk as identified by the GNRI and CONUT scores.

The GNRI and CONUT were used to assess the risk of malnutrition. A total of 503 (62.41%) patients were at risk of malnutrition. Among them, 347 (43.05%) cases were identified by GNRI, 356 (44.17%) cases by CONUT, and 200 (24.81%) patients by both GNRI and CONUT (Figure 2A). A total of 133 (16.50%) patients were at risk of moderate to severe malnutrition. Of them, 109 (13.52%) and 64 (7.94%) patients were at moderate to severe risk of malnutrition according to GNRI and CONUT scores, respectively (Figure 2B). Notably, as assessed by both GNRI and CONUT scores, 40 (4.96%) patients were at moderate to severe risk of malnutrition.

Based on the GNRI score, patients with malnutrition risk, when compared to those without malnutrition risk, were older and had lower BMI, lymphocyte count, total cholesterol, triglycerides, low-density lipoprotein, high-density lipoprotein, albumin, and estimated glomerular filtration rate (Supplementary Table 2). Moreover, this group consisted of more males, had higher proportions of patients with hypertension, history of stroke, and smokers, as well as higher neutrophil-to-lymphocyte ratio, and showed higher grades for total WMH, PWMH, DWMH, total PVS, BG-PVS, CSO-PVS, and more lacune and CMB. Similarly, patients at risk of malnutrition by CONUT were older and demonstrated higher systolic blood pressure, but lower BMI, lymphocyte count, total cholesterol, triglycerides, low-density lipoprotein, high-density lipoprotein, albumin, and estimated glomerular filtration rate. Additionally, they were more likely to be male and there were higher proportions of patients with hypertension, diabetes, coronary heart disease, history of stroke, and medicine use. This group also displayed a higher neutrophil-to-lymphocyte ratio and higher grades for total WMH, PWMH, DWMH, total PVS, BG-PVS, CSO-PVS, and more lacune and CMB (Supplementary Table 2).

**Figure 3:**
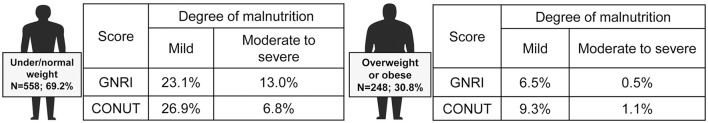
Prevalence of malnutrition in different subgroups of patients according to BMI. Based on body mass index (BMI), patients were divided into underweight (<18.5 kg/m^2^), normal weight (18.5–24.9 kg/m^2^), overweight (25.0–29.9 kg/m^2^), and obese (≥30 kg/m^2^).

The analysis based on BMI classification showed that among patients with normal or low BMI, 23.1% had a mild risk of malnutrition and 13.0% had a moderate to severe risk according to the GNRI score, while based on the CONUT score, 26.9% had a mild risk and 6.8% had a moderate to severe risk (Figure 3). In contrast, among overweight or obese patients, 6.5% were at risk for mild malnutrition and 0.5% for moderate to severe malnutrition based on the GNRI score, whereas based on the CONUT score, 9.3% were at risk for mild malnutrition and 1.1% for moderate to severe malnutrition.

### 3.3 Association between malnutrition risk and CSVD

**Figure 4:**
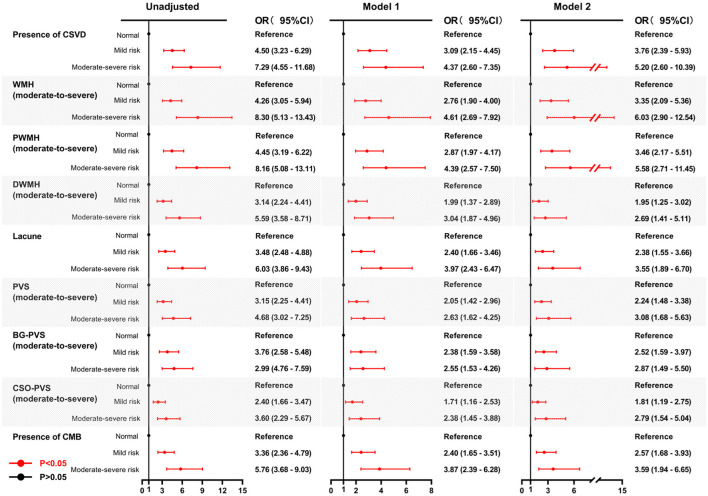
Forest plots for the association between malnutrition risk, as assessed by GNRI, and CSVD along with its common imaging markers. Two multivariable logistic regression models were constructed. Model 1: the covariates of age and sex were adjusted. Model 2: The following covariates were adjusted: age, sex, hypertension, diabetes, coronary heart disease, history of stroke, dyslipidemia, smoking, drinking, body mass index, systolic blood pressure, neutrophil-to-lymphocyte ratio, hemoglobinA1c, homocysteine, estimated glomerular filtration rate, and medication use (antiplatelets and statins). GNRI, geriatric nutritional risk index; CSVD, cerebral small vessel disease; OR, odds ratio; CI, confidence interval; WMH, white matter hyperintensity; DWMH, deep white matter hyperintensity; PWMH, periventricular white matter hyperintensity; PVS, perivascular spaces; BG-PVS, basal ganglia perivascular spaces; CSO-PVS, centrum semiovale perivascular spaces; CMB, cerebral microbleed.

**Figure 5:**
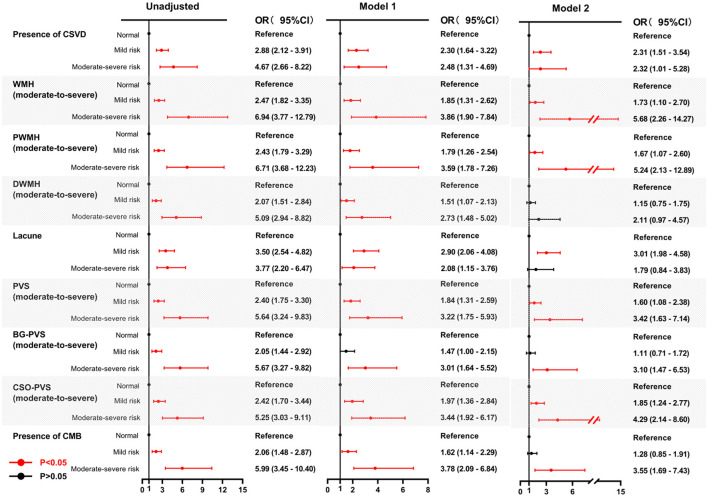
Forest plots for the association between malnutrition risk, as assessed by CONUT, and CSVD along its common imaging markers. Two multivariable logistic regression models were constructed. Model 1: the covariates of age and sex were adjusted. Model 2: The following covariates were adjusted: age, sex, hypertension, diabetes, coronary heart disease, history of stroke, dyslipidemia, smoking, drinking, body mass index, systolic blood pressure, neutrophil-to-lymphocyte ratio, hemoglobinA1c, homocysteine, estimated glomerular filtration rate, and medication use (antiplatelets and statins). CONUT, controlling nutritional status; CSVD, cerebral small vessel disease; OR, odds ratio; CI, confidence interval; WMH, white matter hyperintensity; DWMH, deep white matter hyperintensity; PWMH, periventricular white matter hyperintensity; PVS, perivascular spaces; BG-PVS, basal ganglia perivascular spaces; CSO-PVS, centrum semiovale perivascular spaces; CMB, cerebral microbleed.

Univariate analysis revealed significant associations between both mild and moderate-to-severe malnutrition risk, assessed by either the GNRI or the CONUT, and the presence of CSVD along with its corresponding imaging markers. The association between malnutrition risk, as defined by GNRI scores, and the presence of CSVD and its imaging markers remained significant after adjusting for age and sex (Model 1) (Figure 4). Similarly, using the CONUT score, the mild and moderate-to-severe malnutrition risk demonstrated a significant association with the presence of CSVD and its imaging markers, except for mild malnutrition risk, which was not associated with BG-PVS (Model 1) (Figure 5). Following further adjustments for factors not included in the scoring system or other clinical considerations (Model 2), the association between malnutrition risk, as determined by the GNRI score, and the presence of CSVD and its imaging markers remained significant (Figure 4). However, no significant associations were identified between malnutrition risk and DWMH, moderate to severe malnutrition risk and lacunes, or mild malnutrition risk and the presence of CMB, as defined by the CONUT score (Model 2) (Figure 5).

### 3.4 Subgroup and interaction analyses of the relationship between GNRI and CONUT scores and the presence of CSVD

**Figure 6:**
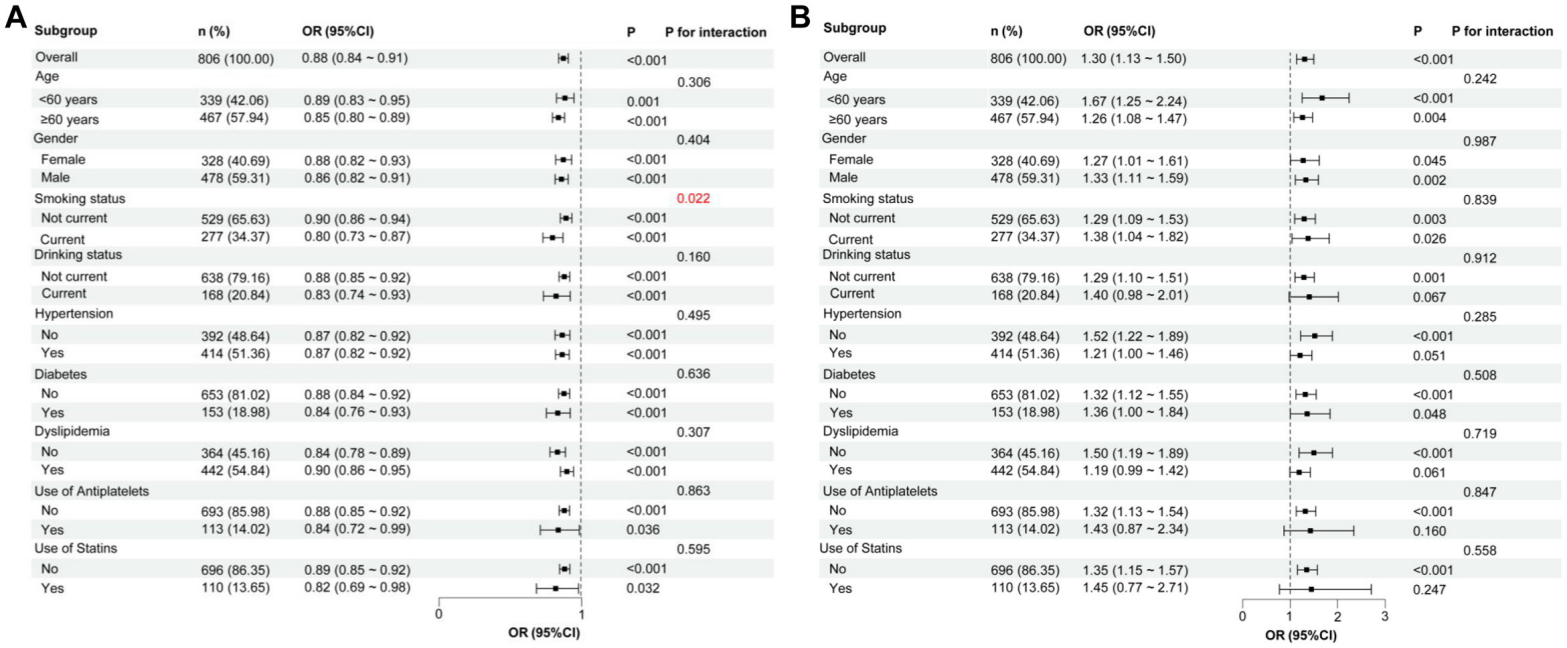
Subgroup analyses of the relationship between GNRI, CONUT, and the presence of CSVD. **(A)** GNRI score and the presence of CSVD. **(B)** CONUT score and the presence of CSVD. In each subgroup analysis, the following factors were adjusted, including age, sex, hypertension, diabetes, coronary heart disease, history of stroke, dyslipidemia, smoking, drinking, body mass index, systolic blood pressure, neutrophil-to-lymphocyte ratio, hemoglobinA1c, homocysteine, estimated glomerular filtration rate, and medication use (antiplatelets and statins). CSVD, cerebral small vessel disease; GNRI, geriatric nutritional risk index; CONUT, controlling nutritional status; OR, odds ratio; CI, confidence interval.

To determine whether the impact of malnutrition risk was affected by other factors, we performed subgroup and interaction analyses on the relationship between the GNRI and CONUT scores and the presence of CSVD. As shown in Figure 6A, significant associations were observed between the GNRI score and the presence of CSVD across all subgroups (*P* < 0.05). However, there was no significant association between the CONUT score and CSVD in the subgroups with current drinking, hypertension, dyslipidemia, and medication use (antiplatelets and statins) (Figure 6B). Notably, there were significant associations of GNRI and CONUT scores with CSVD in the age subgroups of ≥60 years and <60 years (Figures 6A, B). After adjusting for confounding variables, an interaction effect between GNRI score and smoking on the presence of CSVD was observed (Figure 6A). However, no such interaction was found between the CONUT score and the presence of CSVD (Figure 6B).

## 4 Discussion

In this study, we evaluated the risk of malnutrition upon admission in patients from the neurology department by utilizing objective malnutrition scores, namely the GNRI and CONUT. Additionally, we explored the relationship between malnutrition risk and CSVD and its imaging markers. Our results indicated that individuals at risk of malnutrition exhibited a more severe CSVD compared to those not at risk. Moreover, an increased risk of malnutrition correlated with increased severity of imaging markers of CSVD, even after adjusting for confounding variables.

In the study cohort, 43.05% of participants were identified as being at risk of malnutrition based on the GNRI definition, while 44.17% were classified as at risk by the CONUT definition. This indicates a marginal difference in assessing malnutrition between GNRI and CONUT. Interestingly, even in individuals classified as overweight or obese, 7.0% scored as malnourished according to the GNRI, and 10.4% according to the CONUT criteria. These results suggest a potential risk of malnutrition among patients with elevated BMI, challenging common perceptions. This phenomenon is recognized as the double burden of malnutrition (22). Given the rapid global nutrition transition, an increasing number of individuals are experiencing diverse forms of malnutrition throughout their lives, bearing the dual burden directly. Malnutrition and overweight exhibit intricate physiological connections and interactions (23), underscoring the importance of addressing malnutrition concerns in overweight or obese patients.

There are still few studies on the association between malnutrition risk or malnutrition and CSVD, and only a small number of studies on WMH have been reported (24, 25). In this study, we found that malnutrition risk based on GNRI and CONUT assessment was associated with CSVD and its common imaging markers, suggesting that malnutrition may be a potential risk factor for CSVD. However, the mechanisms underlying this association remain unclear. We speculate several possible causes. First, malnutrition may be induced by inflammation and may trigger inflammation (26, 27). Inflammation leads to decreased appetite and increased muscle and lipolysis, exacerbating malnutrition by upregulating the expression of pro-inflammatory factors such as tumor necrosis factor-α, monocyte chemoattractant protein-1, and IL-6 (28, 29). Notably, the rapid turnover of immune cells also requires nutrient supply (28), and malnutrition adversely affects both innate and adaptive immunity (30, 31), thereby increasing susceptibility to infections. Second, altered nutritional status has also been associated with increased oxidative stress (27, 32). Malnutrition increases levels of hydroxynonenal and malondialdehyde (33), which serve as circulating markers of oxidative stress that can lead to structural protein changes, loss of enzyme activity, DNA damage, and apoptosis (34–36). Oxidative stress is negatively correlated with nutritional status in elderly populations (37) and contributes to vascular endothelial injury and dysfunction (38, 39). Furthermore, oxidative stress and inflammation are known to interact; circulating oxidative metabolites can activate inflammatory signaling pathways, while inflammation can promote oxidative stress (40). Thus, there may be a complex interaction between malnutrition, inflammation, and oxidative stress. The pathogenesis of CSVD involves both inflammation and oxidative stress (39, 41), suggesting that malnutrition may exacerbate the development of CSVD through these mechanisms. Third, both GNRI and CONUT scores include serum albumin as an indicator. Albumin is an important protein affecting the physiological function of the circulatory system and has physiological characteristics such as anti-inflammation, anti-oxidation, and anti-thrombosis (42). Furthermore, albumin levels are also closely related to cardiovascular and cerebrovascular diseases (43, 44). Albumin plays a vital role in maintaining capillary membrane stability and fluid balance (45) and provides protective effects against endothelial dysfunction caused by inflammation and oxidative stress (46). Given that endothelial dysfunction in small vessels is a significant factor in CSVD pathogenesis (47), this may further elucidate the association between malnutrition risk, as assessed by the GNRI and CONUT, and CSVD.

Our further analysis showed that CONUT-defined malnutrition risk was associated with PWMH but not with DWMH. In contrast, GNRI-defined malnutrition risk was associated with both PWMH and DWMH. We believe this difference is mainly due to the inclusion of different indicators in the two scoring systems. Specifically, the lymphocyte and cholesterol measures are included in the CONUT score. Inflammation is associated with PWMH but not DWMH (41), while lymphocytes as inflammatory markers may contribute to this difference. PWMH is more affected by hypotension, hypoperfusion, and atrophy (48), whereas DWMH is more susceptible to arteriolosclerosis (49). Cholesterol plays a crucial role in the formation and maintenance of new synapses in the central nervous system (50) and helps buffer brain tissue against hypoxia following cerebral ischemia (51). Therefore, low cholesterol may mitigate DWMH and aggravate PWMH. The association between higher CONUT scores and lower cholesterol levels may explain why CONUT is associated with PWMH rather than DWMH.

The association of malnutrition or malnutrition risk with lacune, PVS, and CMB has not been previously reported. Here, we found that the risk of malnutrition was significantly associated with lacune, PVS (including BG-PVS and CSO-PVS), and CMB. The underlying mechanisms may be related to the aforementioned factors of inflammation, oxidative stress, and endothelial cell dysfunction. Notably, BG-PVS and CMB were only associated with the risk of moderate-severe malnutrition as defined by CONUT, but not with the risk of mild malnutrition. This may suggest that a higher degree of malnutrition is required to exhibit an association with these imaging markers. Lacune, however, was associated with mild malnutrition risk as defined by CONUT, but not with moderate-severe malnutrition risk. This may be due to the inclusion of cholesterol in the CONUT score. Most lacunes are attributed to small subcortical infarcts (i.e., lacunar ischemic strokes) (52), and high cholesterol is associated with an increased risk of such strokes (53). Higher CONUT scores, which reflect lower cholesterol levels, may influence the association of other indicators within CONUT, potentially weakening the relationship between moderate to severe malnutrition risk and lacunes. Nonetheless, this finding should be interpreted cautiously, potentially due to sample size limitations. Additionally, in the subgroup analysis, we identified an interaction between the GNRI score and smoking on the presence of CSVD. Consistently, it has been proposed that nicotine in tobacco could potentially decrease food consumption (54), which might exacerbate malnutrition. No significant association between CONUT and CSVD was observed in the subgroup with current drinking, which may be due to the small sample size and requires further study validation. Hypertension, particularly in its chronic and severe forms, plays a critical role in cerebrovascular dynamics (55, 56). In this study, the continuous variable of systolic blood pressure was adjusted as a confounding variable alongside the categorical variable for hypertension (presence/absence). We found that there was a lack of association between CONUT and CSVD specifically in patients with hypertension and dyslipidemia. This may be because hypertensive patients often have dyslipidemia (57), and high cholesterol decreases the CONUT score. Additionally, there are conflicting relationships between medication use, including combination therapies involving multiple drugs, and malnutrition (58). Some scholars have suggested avoiding antiplatelet drugs in occult CSVD and do not support the use of lipid-lowering drugs (47), and there are also views that antiplatelet drugs and lipid-lowering drugs have contradictory effects on WMH (59). In this study, we found that CONUT lacked an association with CSVD in the subgroup taking antiplatelets and statins, indicating that these drugs may offer some protective effects against malnutrition in CSVD. However, this was not observed in the malnutrition risk defined by GNRI scores.

Our study has several limitations. Firstly, due to the unavailability of necessary variables, we were unable to assess the malnutrition status using the ESPEN and GLIM diagnostic criteria and consequently could not compare them with the GNRI and CONUT scores. Secondly, other common imaging markers of CSVD (e.g., recent subcortical small infarcts) were not analyzed in this study. Thirdly, due to the retrospective nature of our study, we were unable to collect data on participants' income levels and other socioeconomic indicators, which limits our ability to fully assess how these factors may have influenced the observed relationships between malnutrition and CSVD. Future studies should aim to incorporate comprehensive socioeconomic assessments to better delineate the role of economic and social factors in the prevalence of malnutrition, thus enhancing the understanding of its impact on cognitive and vascular health. Fourthly, certain classes of antihypertensive drugs exhibit adverse patterns of cerebral blood flow regulation (56). However, due to limitations in our data collection process, specific classes of antihypertensive medications used by participants were not systematically recorded. Further studies are needed to understand their potential effects on CSVD. Lastly, this study is a single-center, retrospective data analysis, and thus a causal relationship cannot be established. Prospective studies are needed to confirm our findings.

This study suggests an association between malnutrition risk (measured by objective nutritional indices GNRI and CONUT) and CSVD along with its common imaging markers. Our findings imply that nutritional interventions may prevent the progression of CSVD. Moreover, our findings underscore the potential for using imaging markers of CSVD as indicators for assessing malnutrition risk. As such, routine screening for malnutrition should be considered in clinical settings where patients present with CSVD. We propose that healthcare providers implement nutritional assessments and develop targeted intervention strategies, such as dietary modification or nutritional supplements, to address identified risks. Future research should focus on evaluating the effectiveness of these interventions on clinical outcomes for patients with CSVD.

## Data Availability

The raw data supporting the conclusions of this article will be made available by the authors, without undue reservation.
