# Supplementary material for: Association between cerebral small vessel disease and malnutrition risk: a retrospective cross-sectional study
Source: Front Neurol. 2025 Jun 18;16:1512109. doi: 10.3389/fneur.2025.1512109 (PMC12213786; doi:10.3389/fneur.2025.1512109)
Supplement: Supplementary file 1 [file Table_1.docx]

Supplementary Material

# Supplementary Tables

**Supplemental Table 1.** Baseline characteristics of patients in the non-CSVD group and CSVD group based on the total CSVD score.

| **Variables** | **Overall**  **(N = 806)** | **Non-CSVD group**  **(n = 450)** | **CSVD group (n = 356)** | ***P* value** |
| --- | --- | --- | --- | --- |
| **Demographic data** | | | | |
| Age, median (IQR) | 63 (53, 71) | 57 (49, 66) | 68 (60, 74) | < 0.001 |
| Male, n (%) | 478 (59.31) | 229 (50.89) | 249 (69.94) | < 0.001 |
| **Risk factors, n (%)** | | | | |
| Hypertension | 414 (51.36) | 170 (37.78) | 244 (68.54) | < 0.001 |
| Diabetes | 153 (18.98) | 63 (14.00) | 90 (25.28) | < 0.001 |
| Coronary heart disease | 48 (5.96) | 17 (3.78) | 31 (8.71) | 0.003 |
| History of stroke | 111 (13.77) | 14 (3.11) | 97 (27.25) | < 0.001 |
| Dyslipidemia | 442 (54.84) | 220 (48.89) | 222 (62.36) | < 0.001 |
| Current smoking | 277 (34.37) | 112 (24.89) | 165 (46.35) | < 0.001 |
| Current drinking | 168 (20.84) | 85 (18.89) | 83 (23.31) | 0.125 |
| BMI, kg/m^2^ (median, IQR) | 23.53 (21.26, 25.76) | 23.73 (21.60, 26.57) | 23.34 (20.76, 24.86) | < 0.001 |
| **Nutritional status** | | | | |
| GNRI, continuous (median, IQR) | 99.32 (95.16, 103.34) | 101.86 (97.84, 105.28) | 96.40 (92.47, 99.47) | <0.001 |
| GNRI categories, (n, %) |  |  |  | < 0.001 |
| Normal | 459 (56.95) | 333 (74.00) | 126 (35.39) |  |
| Mild risk | 238 (29.53) | 88 (19.56) | 150 (42.13) |  |
| Moderate-severe risk | 109 (13.52) | 29 (6.44) | 80 (22.47) |  |
| CONUT, continuous (median, IQR) | 1 (0, 2) | 1 (0, 2) | 2 (1, 3) | <0.001 |
| CONUT categories, (n, %) |  |  |  | <0.001 |
| Normal | 450 (55.83) | 306 (68.00) | 144 (40.45) |  |
| Mild risk | 292 (36.23) | 124 (27.56) | 168 (47.19) |  |
| Moderate-severe risk | 64 (7.94) | 20 (4.44) | 44 (12.36) |  |
| **Clinical and laboratory features, mean ± SD or median (IQR)** | | | | |
| Systolic blood pressure, mmHg | 138 (124, 153) | 131 (119, 144) | 145 (132, 166) | <0.001 |
| Diastolic blood pressure, mmHg | 85 (76, 94) | 81 (74, 90) | 88 (78, 98) | <0.001 |
| Neutrophil count, × 10 ^9^/L | 4.43 (3.36, 6.29) | 4.23 (3.26, 6.16) | 4.69 (3.58, 6.46) | 0.007 |
| Lymphocyte count, × 10 ^9^/L | 1.69 (1.25, 2.22) | 1.81 (1.34, 2.37) | 1.54 (1.15, 1.94) | <0.001 |
| NLR | 2.63 (1.71, 4.37) | 2.30 (1.57, 3.94) | 2.97 (2.03, 4.60) | <0.001 |
| Total cholesterol, mmol/L | 4.92 ± 1.30 | 5.15 ± 1.24 | 4.64 ± 1.33 | <0.001 |
| Triglycerides, mmol/L | 1.23 (0.90, 1.88) | 1.27 (0.90, 2.00) | 1.20 (0.89, 1.65) | 0.018 |
| Low-density lipoprotein, mmol/L | 3.18 ± 0.96 | 3.32 ± 0.91 | 3.00 ± 0.99 | <0.001 |
| High-density lipoprotein, mmol/L | 1.12 (0.92, 1.35) | 1.20 (1.02, 1.46) | 1.01 (0.84, 1.19) | <0.001 |
| Albumin, g/l | 39.37 ± 3.88 | 40.85 ± 3.63 | 37.49 ± 3.35 | <0.001 |
| HbA1c, % | 6.00 (5.70, 6.50) | 5.90 (5.67, 6.40) | 6.08 (5.70, 6.66) | <0.001 |
| Homocysteine, mmol/L | 10.20 (8.10, 13.10) | 9.40 (7.60, 11.30) | 12.10 (9.50, 15.33) | <0.001 |
| Estimated glomerular filtration rate, ml/min | 96.46 (84.06, 105.10) | 99.15 (89.08, 107.81) | 91.38 (77.26, 102.25) | <0.001 |
| **Concomitant medications, n (%)** | | | | |
| Antiplatelets | 113 (14.02) | 41 (9.11) | 72 (20.22) | <0.001 |
| Statins | 110 (13.65) | 46 (10.22) | 64 (17.98) | 0.001 |

Abbreviations: CSVD, cerebral small vessel disease, GNRI, geriatric nutritional risk index; CONUT, controlling nutritional status; BMI, body mass index; NLR, neutrophil-to-lymphocyte ratio; HbA1c, hemoglobinA1c. IQR, interquartile range; SD, standard deviation.

**Supplemental Table 2.** Baseline characteristics of patients with or without malnutrition based on GNRI and CONUT scores.

| Variables | GNRI | | | CONUT | | |
| --- | --- | --- | --- | --- | --- | --- |
|  | No malnutrition  (n = 459) | Malnutrition  (n = 347) | P value | No malnutrition  (n = 450) | Malnutrition  (n = 356) | P value |
| **Demographic data** | | | | | | |
| Age, median (IQR) | 58 (50, 66) | 68 (59, 74) | < 0.001 | 59 (52, 68) | 67 (57, 74) | < 0.001 |
| Male, n (%) | 256 (55.77) | 222 (63.98) | 0.019 | 252 (56.00) | 226 (63.48) | 0.032 |
| **Risk factors, n (%)** | | | | | | |
| Hypertension | 221 (48.15) | 193 (55.62) | 0.036 | 198 (44.00) | 216 (60.67) | < 0.001 |
| Diabetes | 82 (17.86) | 71 (20.46) | 0.352 | 69 (15.33) | 84 (23.60) | 0.003 |
| Coronary heart disease | 26 (5.66) | 22 (6.34) | 0.688 | 18 (4.00) | 30 (8.43) | 0.008 |
| History of stroke | 44 (9.59) | 67 (19.31) | < 0.001 | 33 (7.33) | 78 (21.91) | < 0.001 |
| Dyslipidemia | 245 (53.38) | 197 (56.77) | 0.338 | 250 (55.56) | 192 (53.93) | 0.646 |
| Current smoking | 141 (30.72) | 136 (39.19) | 0.012 | 155 (34.44) | 122 (34.27) | 0.959 |
| Current drinking | 96 (20.92) | 72 (20.75) | 0.954 | 101 (22.44) | 67 (18.82) | 0.208 |
| BMI, kg/m^2^ (median, IQR) | 24.38 (22.80, 26.84) | 21.79 (19.72, 24.22) | <0.001 | 23.94 (21.64, 26.33) | 23.04 (20.75, 24.89) | < 0.001 |
| **Nutritional status** | | | | | | |
| GNRI (median, IQR) | 102.60 (100.53, 106.02) | 94.11 (90.93, 96.50) | <0.001 | 101.11 (97.13, 104.09) | 96.93 (91.69, 100.96) | < 0.001 |
| CONUT (median, IQR) | 1 (0, 2) | 2 (1, 4) | <0.001 | 0 (0, 1) | 3 (2, 4) | < 0.001 |
| **Clinical and laboratory features, mean ± SD or median (IQR)** | | | | | | |
| Systolic blood pressure, mmHg | 138 (124, 152) | 138 (124, 155) | 0.648 | 135 (121, 151) | 140 (127, 156) | 0.011 |
| Diastolic blood pressure, mmHg | 85 (77, 95) | 83 (75, 93) | 0.087 | 85 (76, 95) | 85 (75, 93) | 0.255 |
| Neutrophil count, × 10 ^9^/L | 4.32 (3.33, 6.06) | 4.55 (3.48, 6.49) | 0.233 | 4.19 (3.33, 5.54) | 4.96 (3.44, 6.98) | <0.001 |
| Lymphocyte count, × 10 ^9^/L | 1.78 (1.30, 2.35) | 1.58 (1.16, 2.05) | <0.001 | 1.98 (1.65, 2.45) | 1.19 (0.94, 1.56) | <0.001 |
| NLR | 2.45 (1.61, 4.04) | 2.96 (1.91, 4.49) | <0.001 | 2.12 (1.55, 3.00) | 3.94 (2.42, 6.46) | <0.001 |
| Total cholesterol, mmol/L | 5.15 ± 1.25 | 4.62 ± 1.32 | <0.001 | 5.33 (4.63, 6.12) | 4.08 (3.36, 4.89) | <0.001 |
| Triglycerides, mmol/L | 1.41 (1.00, 2.07) | 1.07 (0.79, 1.50) | <0.001 | 1.43 (1.01, 2.10) | 1.06 (0.77, 1.47) | <0.001 |
| Low-density lipoprotein, mmol/L | 3.34 (2.69, 3.91) | 2.97 (2.25, 3.56) | <0.001 | 3.50 (2.98, 4.02) | 2.59 (2.03, 3.26) | <0.001 |
| High-density lipoprotein, mmol/L | 1.17 (0.98, 1.40) | 1.06 (0.86, 1.23) | <0.001 | 1.17 (0.99, 1.40) | 1.06 (0.86, 1.25) | <0.001 |
| Albumin, g/l | 41.40 (39.80, 43.70) | 36.60 (34.75, 37.70) | <0.001 | 40.30 (38.10, 42.50) | 37.90 (34.80, 40.73) | <0.001 |
| HbA1c, % | 6.00 (5.70, 6.50) | 6.00 (5.67, 6.50) | 0.703 | 6.00 (5.70, 6.50) | 6.00 (5.66, 6.50) | 0.339 |
| Homocysteine, mmol/L | 9.70 (7.80, 12.25) | 11.10 (8.70, 14.05) | <0.001 | 10.00 (8.00, 12.50) | 10.75 (8.50, 13.70) | 0.003 |
| Estimated glomerular filtration rate, ml/min | 98.47 (88.00, 106.75) | 91.60 (78.59, 102.69) | <0.001 | 97.80 (88.07, 106.03) | 93.80 (79.34, 103.42) | <0.001 |
| **Concomitant medications, n (%)** | | | | | | |
| Antiplatelet | 56 (12.20) | 57 (16.43) | 0.087 | 34 (7.56) | 79 (22.19) | <0.001 |
| Statin | 57 (12.42) | 53 (15.27) | 0.242 | 33 (7.33) | 77 (21.63) | <0.001 |
| **Neuroimaging markers, n (%)** | | | | | | |
| Moderate to severe WMH | 123 (26.80) | 227 (65.42) | < 0.001 | 144 (32.00) | 206 (57.87) | < 0.001 |
| Moderate to severe PWMH | 116 (25.27) | 223 (64.27) | < 0.001 | 139 (30.89) | 200 (56.18) | < 0.001 |
| Moderate to severe DWMH | 96 (20.92) | 173 (49.86) | < 0.001 | 111 (24.67) | 158 (44.38) | < 0.001 |
| Presence of lacune | 96 (20.92) | 181 (52.16) | < 0.001 | 99 (22.00) | 178 (50.00) | < 0.001 |
| Moderate to severe PVS | 101 (22.00) | 174 (50.14) | < 0.001 | 108 (24.00) | 167 (46.91) | < 0.001 |
| Moderate to severe BG-PVS | 61 (13.29) | 133 (38.33) | < 0.001 | 75 (16.67) | 119 (33.43) | < 0.001 |
| Moderate to severe CSO-PVS | 75 (16.34) | 121 (34.87) | < 0.001 | 72 (16.00) | 124 (34.83) | < 0.001 |
| Presence of CMB | 78 (16.99) | 156 (44.96) | < 0.001 | 93 (20.67) | 141 (39.61) | < 0.001 |

Abbreviations: GNRI, geriatric nutritional risk index; CONUT, controlling nutritional status; BMI, body mass index; NLR, neutrophil-to-lymphocyte ratio; HbA1c, hemoglobinA1c; WMH, white matter hyperintensity; PWMH, periventricular white matter hyperintensity; DWMH, deep white matter hyperintensity; PVS, perivascular spaces; BG-PVS, basal ganglia perivascular spaces; CSO-PVS, centrum semiovale perivascular spaces; CMB, cerebral microbleed. IQR, interquartile range; SD, standard deviation.
